# Existence of Classical Solutions for Nonlinear Elliptic Equations with Gradient Terms

**DOI:** 10.3390/e24121829

**Published:** 2022-12-15

**Authors:** Yongxiang Li, Weifeng Ma

**Affiliations:** Department of Mathematics, Northwest Normal University, Lanzhou 730070, China

**Keywords:** elliptic equation, gradient term, classical solution, positive solution, 5J25, 35J60, 47H10

## Abstract

This paper deals with the existence of solutions of the elliptic equation with nonlinear gradient term −Δu=f(x,u,∇u) on Ω restricted by the boundary condition ${u|}_{\partial \Omega }=0$, where Ω is a bounded domain in $R^{N}$ with sufficiently smooth boundary ∂Ω, N≥2, and $f:\bar{\Omega }\times R\times R^{N}\to R$ is continuous. The existence results of classical solutions and positive solutions are obtained under some inequality conditions on the nonlinearity f(x,ξ,η) when |(ξ,η)| is small or large enough.

## 1. Introduction and Main Results

Let Ω be a bounded domain in $R^{N}\left(N\geq 2\right)$ whose boundary ∂Ω is $C^{2+\mu }$-smooth for given μ∈(0,1). In this paper, we discuss the existence of solutions of the elliptic boundary value problem (BVP) with gradient term

(1) $$\left\{\begin{array}{c} -\Delta u=f(x,\;u,\;\nabla u)\;,\;x\in \Omega \;,\;\\ {u|}_{\partial \Omega }=0\;, \end{array}\right.$$

where $f:\bar{\Omega }\times R\times R^{N}\to R$ is the nonlinearity. This problem arises in many different areas of applied mathematics. Due to the appearance of the gradient term in the nonlinearity, BVP(1) has no variational structure, and the variational method and critical point theory cannot be applied to it directly. The authors of [1,2] proposed a method combining the mountain-pass theorem with an approximation technique to solve BVP(1). Firstly, for any given $w\in H_{0}^{1}\left(\Omega \right)$, they considered the boundary value problem

(2) $$\left\{\begin{array}{c} -\Delta u=f(x,\;u,\;\nabla w)\;,\;x\in \Omega \;,\;\\ {u|}_{\partial \Omega }=0\;. \end{array}\right.$$

Note that BVP(2) has the variational structure. They established the existence of a solution $u_{w}$ of BVP(2) by using the mountain-pass theorem. Then, they constructed a sequence $\left\{u_{n}\right\}\subset H_{0}^{1}\left(\Omega \right)$ by the iterative equation

(3) $$\left\{\begin{array}{c} -\Delta u_{n}=f\left(x,\;u_{n},\;\nabla u_{n-1}\right)\;,\;x\in \Omega \;,\;\\ u_{n}{|}_{\partial \Omega }=0 \end{array}\right.$$

starting with an arbitrary $u_{0}\in H_{0}^{1}\left(\Omega \right)\cap C^{1}\left(\bar{\Omega }\right)$, and they proved that $\left\{u_{n}\right\}$ converges to a solution of BVP(1) in that f(x,ξ,η) satisfies Lipschitz conditions on (ξ,η) in the neighborhood of (0,0) with appropriately small coefficients and certain growth conditions on ξ. Later, this iterative method based on the mountain-pass theorem was applied to many semilinear and quasilinear elliptic equations; see [3,4,5,6,7]. In [8], Ruiz obtained the existence of a positive solution for BVP(1) by combining Krasnoselskii’s fixed-point theorem in cones with blow-up techniques when f(x,ξ,η) is a nonnegative function and satisfies a suitable growth condition on ξ and η. When Ω is a ball, annulus, or exterior domain of a ball, and f(x,ξ,η) is radially symmetric on *x*, the authors of [9,10,11,12,13] obtained the existence of positive radial solutions of BVP(1) by discussing the corresponding boundary value problem of second-order ordinary differential equations.

On the other hand, the lower- and upper-solutions method is an effective way to obtain the existence of solutions of BVP(1). In [14], Amann built a lower- and upper-solution theorem of BVP(1) in $C^{2+\mu }\left(\bar{\Omega }\right)$ in that f(x,ξ,η) has a continuous partial derivative with respect to ξ and η, and there is, at most, quadratic growth on η. He assumed BVP(1) has pair of ordered lower and upper solutions and proved the existence of a solution between the lower and upper solutions. In [15], Amann and Crandall slightly generalized the results of [14] by a more-direct argument. In [16], Pohozaev obtained the existence results for BVP(1) via the method of lower and upper solutions in the Sobolev space $W^{2,p}\left(\Omega \right)$ with p>N when f(x,ξ,η) is Lipschitzian with respect to η. In [17,18,19,20,21,22], the authors obtained the existence of solutions or positive solutions by using the lower- and upper-solutions method and fixed-point theorem under some growth condition of the nonlinearity.

In this paper, we apply the upper- and lower-solution method and the Leray–Schauder fixed-point theory to obtain new existence results. In the following, we denote a generic point of $\bar{\Omega }\times R\times R^{N}$ by (x,ξη) with $x\in \bar{\Omega }$, ξ∈R, and $\eta =\left(\eta_{1},\cdots ,\eta_{N}\right)\in R^{N}$. To obtain the classical solution of BVP(1), we assume the nonlinearity $f:\bar{\Omega }\times R\times R^{N}\to R$ satisfies the following conditions:

(F1) The partial derivatives $f_{\xi }^{\prime }$, $f_{\eta_{i}}^{\prime },\;i=1,\;\cdots ,\;N$, exist and are continuous on $\bar{\Omega }\times R\times R^{N}$, and for every ρ>0, there exists L:=L(ρ)>0 such that
  (4) $$|f\left(x_{2},\;\xi ,\eta \right)-f\left(x_{1},\;\xi ,\eta \right)\left|\leq L\right|x_{2}-x_{1}{|}^{\mu },$$
  for any $x_{1},\;x_{2},\in \bar{\Omega }$, ξ∈[−ρ,ρ] and $\eta_{2}\in {\bar{B}}_{\rho }:=\{\eta \in R^{N}\left|\;|\eta |\leq \rho \right\}$.
(F2) For every ρ>0, there exists C:=C(ρ)>0 such that
  (5) $$|f\left(x,\;\xi ,\eta \right)|\leq C(1+|\eta {|}^{2}),\;$$
  for any $\left(x,\;\xi ,\eta \right)\in \bar{\Omega }\times \left[-\rho ,\;\rho \right]\times R^{N}$.

Condition (F1) implies that *f* is continuous on $\bar{\Omega }\times R\times R^{N}$ and is a stronger regularity condition. Condition (F2) restricts *f* to at most quadratic growth with respect to η. If *f* grows at most like ${\left|\eta \right|}^{2-\varepsilon }$ for some ε∈(0,1), the regularity condition (F1) can be weakened as

(F1)′ For every ρ>0, there exists L:=L(ρ)>0 such that
  $$|f\left(x_{2},\;\xi_{2},\eta_{2}\right)-f\left(x_{1},\;\xi_{1},\eta_{1}\right)\left|\leq L(\right|x_{2}-x_{1}{|}^{\mu }+|\xi_{2}-\xi_{1}\left|+\right|\eta_{2}-\eta_{1}\left|\right),$$
  for any $\left(x_{1},\;\xi_{1},\eta_{1}\right)$, $\left(x_{2},\;\xi_{2},\eta_{2}\right)\in \bar{\Omega }\times \left[-\rho ,\;\rho \right]\times {\bar{B}}_{\rho }$.

See [3,14].

Our existence results are related to the principle eigenvalue $\lambda_{1}$ of Laplace operator −Δ on the boundary condition ${u|}_{\partial \Omega }=0$, which is given by

(6) $$\lambda_{1}=\inf\left\{\frac{{∥\nabla u∥}_{2}}{{∥u∥}_{2}}\;|\;u\in H_{0}^{1}\left(\Omega \right),\;{∥u∥}_{2}\neq 0\right\}.$$

**Theorem** **1.** *Let $f:\bar{\Omega }\times R\times R^{N}\to R$ satisfy* (F1) *and* (F2)*. If there exist constants a,b≥0 satisfying*

(7) $$\frac{a}{\lambda_{1}}+\frac{b}{\sqrt{\lambda_{1}}}<1$$

*and H>0 such that*

(8) $$f\left(x,\;\xi ,\;\eta \right)\leq a\xi +b|\eta |,\;x\in \bar{\Omega },\;\;\xi \geq 0,\;\;\left|\left(\xi ,\;\eta \right)\right|\geq H$$

*and*

(9) $$f\left(x,\;-\xi ,\;-\eta \right)\geq -a\xi -b|\eta |,\;x\in \bar{\Omega },\;\;\xi \geq 0,\;\;\left|\left(\xi ,\;\eta \right)\right|\geq H,$$

*then, BVP(1) has at least one classical solution $u\in C^{2+\mu }\left(\bar{\Omega }\right)$.*

In Theorem 1, if b=0, the result is known (see [1, Theorem 1.2]), and if b≠0, the result is new.

**Theorem** **2.** *Let $f:\bar{\Omega }\times R\times R^{N}\to R$ satisfy* (F1) *and* (F2)*. If there exist constants a,b≥0 satisfying (7) and H>0 such that f satisfies (8), and there exists a positive constant δ such that*

(10) $$f\left(x,\;\xi ,\;\eta \right)\geq \lambda_{1}\xi ,\;x\in \bar{\Omega },\;\;\xi \geq 0,\;\;\left|\left(\xi ,\;\eta \right)\right|\leq \delta ,$$

*then, BVP(1) has at least one classical positive solution $u\in C^{2+\mu }\left(\bar{\Omega }\right)$.*

If *f* satisfies the condition of Theorem 1, but assume that

(11) $$f\left(x,\;0,\;0\right)\geq 0,\;x\in \bar{\Omega }$$

instead of (9), then $v_{0}\equiv 0$ is a lower solution of BVP(1), and BVP(1) has at least one nonnegative solution, see [1, Theorem 1.3]. Theorem 2 is an addition of this result and uses (10) instead of (11) to obtain a positive solution of BVP(1).

**Theorem** **3.** *Let the conditions of Theorem 1 be satisfied, and there exists a positive constant δ such that (10) and*

(12) $$f\left(x,\;-\xi ,\;-\eta \right)\leq -\lambda_{1}\xi ,\;x\in \bar{\Omega },\;\;\xi \geq 0,\;\;\left|\left(\xi ,\;\eta \right)\right|\leq \delta ,$$

*hold. Then, BVP(1) has at least one positive solution $u_{1}\in C^{2+\mu }\left(\bar{\Omega }\right)$ and one negative solution $u_{2}\in C^{2+\mu }\left(\bar{\Omega }\right)$.*

In Theorem 3, from (10) and (12), it follows that f(x,0,0)≡0 by letting |(ξ,η)|→0. Hence, $u_{3}\equiv 0$ is a trivial solution. This means that BVP(1) has at least three distinct solutions.

The proofs of Theorems 1–3 are based on the method of lower and upper solutions built by Amann [14]. A lower solution *v* of BVP(1) means that $v\in C^{2+\mu }\left(\bar{\Omega }\right)$ and satisfies

$$\left\{\begin{array}{c} -\Delta v\leq f(x,\;v,\;\nabla v)\;,\;x\in \Omega \;,\;\\ {u|}_{\partial \Omega }\leq 0\;, \end{array}\right.$$

and an upper solution *w* of BVP(1) means that $w\in C^{2+\mu }\left(\bar{\Omega }\right)$ and satisfies

$$\left\{\begin{array}{c} -\Delta w\geq f(x,\;w,\;\nabla w)\;,\;x\in \Omega \;,\;\\ {w|}_{\partial \Omega }\geq 0\;. \end{array}\right.$$

By [1, Theorem 1.1], we have the following existence result:

**Theorem** **4.** *Let $f:\bar{\Omega }\times R\times R^{N}\to R$ satisfy* (F1) *and* (F2)*. If BVP(1) has a lower solution $v_{0}$ and an upper solution $w_{0}$ such that $v_{0}\leq w_{0}$, then BVP(1) has at least one solution $u_{2}\in C^{2+\mu }\left(\bar{\Omega }\right)$ between $v_{0}$ and $w_{0}$.*

Theorem 4 is a special case of [1, Theorem 1.1]. In Section 3, we use Theorem 4 to prove Theorems 1–3. Some preliminaries to discuss BVP(1) are presented in Section 2.

## 2. Preliminaries

Let $W^{m,p}\left(\Omega \right)$ be the usual Sobolev space on domain Ω and $H^{m}\left(\Omega \right):=W^{m,2}\left(\Omega \right)$. To discuss BVP(1), we first consider the corresponding linear elliptic boundary value problem (LBVP)

(13) $$\left\{\begin{array}{c} -\Delta u=h(x)\;,\;x\in \Omega \;,\;\\ {u|}_{\partial \Omega }=0\;. \end{array}\right.$$

where $h\in L^{p}\left(\Omega \right)\left(1<p<+\infty \right)$ is a given function. Since the boundary ∂Ω of Ω is $C^{2+\mu }$-smooth, by the $L^{P}$ theory of linear elliptic equations (see [23]), for every $h\in L^{p}\left(\Omega \right)$, LBVP(13) has a unique strong solution $u:=Sh\in W^{2,p}\left(\Omega \right)\cap W_{0}^{1,p}\left(\Omega \right)$, and the solution operator $S:L^{p}\left(\Omega \right)\to W^{2,p}\left(\Omega \right)$ is a linear bounded operator. Especially when p=2, the solution u=Sh of LBVP(13) satisfies

(14) $${∥u∥}_{2}\leq \frac{1}{\sqrt{\lambda_{1}}\;}{\;∥\nabla u∥}_{2}{,\;∥\nabla u∥}_{2}\leq \frac{1}{\sqrt{\lambda_{1}}\;}\;{∥\Delta u∥}_{2}.$$

In fact, since the Laplace operator $-\Delta :H^{2}\left(\Omega \right)\cap H_{0}^{1}\left(\Omega \right)\subset L^{2}\left(\Omega \right)\to L^{2}\left(\Omega \right)$ is a positive definite operator in $L^{2}\left(\Omega \right)$,

$${{∥\nabla u∥}_{2}}^{2}=\left(-\Delta u,\;u\right)\geq \lambda_{1}\left(u,\;u\right)=\lambda_{1}{{∥u∥}_{2}}^{2}.$$

Hence, the first inequality of (14) holds. Noting ${∥\Delta u∥}_{2}={∥h∥}_{2}$, from Equation (13), it follows that

$${{∥\nabla u∥}_{2}}^{2}=\left(-\Delta u,\;u\right)=\left(h,\;u\right)\leq {∥h∥}_{2}{∥u∥}_{2}={∥\Delta u∥}_{2}{∥u∥}_{2}\leq \frac{1}{\sqrt{\lambda_{1}}\;}{\;∥\Delta u∥}_{2}{∥\nabla u∥}_{2}.$$

Hence, the second inequality of (14) holds.

When $h\in C^{\nu }\left(\bar{\Omega }\right)$ for some ν∈(0,μ], by the Schauder theory of linear elliptic equations (see [23,24]), the solution of LBVP(13) $u=Sh\in C^{2+\nu }\left(\bar{\Omega }\right)$ is a classical solution.

Next, consider the nonlinear elliptic equation BVP(1). We have the following existence result of the classical solution:

**Theorem** **5.** *Let $f:\bar{\Omega }\times R\times R^{N}\to R$ satisfy* (F1)${}^{\prime }$ *and in the following growth condition*

(F3) *Let there exist constants a,b≥0 satisfying (7) and c>0 such that*
  $$\left|f\left(x,\;\xi ,\;\eta \right)\right|\leq a|\xi |+b|\eta |+c,\;\left(x,\;\xi ,\;\eta \right)\in \bar{\Omega }\times R\times R^{N}.$$

*Then, BVP(1) has a unique classical solution $u\in C^{2+\mu }\left(\bar{\Omega }\right)$.*

**Proof.**  We first show that BVP(1) has an $L^{2}$ solution $u_{0}\in H^{2}\left(\Omega \right)\cap H_{0}^{1}\left(\Omega \right)$. Since the solution operator of LBVP(13) $S:L^{2}\left(\Omega \right)\to H^{2}\left(\Omega \right)\cap H_{0}^{1}\left(\Omega \right)$ is a linear bounded operator, by the compactness of the Sobolev embedding $H^{2}\left(\Omega \right)↪H^{1}\left(\Omega \right)$, $S:L^{2}\left(\Omega \right)\to H_{0}^{1}\left(\Omega \right)$ is completely continuous. Define a mapping *F* on $H_{0}^{1}\left(\Omega \right)$ by

(15) $$F\left(u\right)\left(x\right)=f\left(x,\;u\left(x\right),\;\nabla u\left(x\right)\right),\;u\in H_{0}^{1}\left(\Omega \right),\;\;x\in \Omega .$$

By Condition (F3), $F:H_{0}^{1}\left(\Omega \right)\to L^{2}\left(\Omega \right)$ is continuous, and it maps every bounded set of $H_{0}^{1}\left(\Omega \right)$ into a bounded set of $L^{2}\left(\Omega \right)$. Hence, the composite mapping

(16) $$A=S\circ F:H_{0}^{1}\left(\Omega \right)\to H_{0}^{1}\left(\Omega \right)$$

is completely continuous. By the definition of *S*, the strong $L^{2}$ solution of LBVP(13) is equivalent to the fixed point of *A*. We use the Leray–Schauder fixed point theorem [25] to show that *A* has a fixed point. For this, we consider the equation family

(17) u=λAu,0<λ<1,

and show that the set of their solutions is bounded in $H_{0}^{1}\left(\Omega \right)$.

Let $u\in H_{0}^{1}\left(\Omega \right)$ be a solution of (17) for λ∈(0,1). Set h=λF(u). Since $h\in L^{2}\left(\Omega \right)$, by the definition of *S*, $u=Sh\in H^{2}\left(\Omega \right)\cap H_{0}^{1}\left(\Omega \right)$ is the unique solution of LBVP(13). Hence, *u* satisfies the differential equation

(18) $$\left\{\begin{array}{c} -\Delta u=\lambda \;f(x,\;u,\;\nabla u)\;,\;x\in \Omega \;,\;\\ {u|}_{\partial \Omega }=0\;. \end{array}\right.$$

By this equation and Condition (F3), we have

|Δu(x)|=|λf(x,u(x),∇u(x))|≤a|u(x)|+b|∇u(x)|+c,x∈Ω.

By this inequality and (14), we obtain that

$$\begin{array}{ccc} \sqrt{\lambda_{1}}{∥\nabla u∥}_{2}\;\leq \;{∥\Delta u∥}_{2} & \leq & {a∥u∥}_{2}+b{∥\nabla u∥}_{2}+c\sqrt{\left|\Omega \right|}\\ & \leq & \left(\frac{a}{\sqrt{\lambda_{1}}\;}+b\right){∥\nabla u∥}_{2}+c\sqrt{\left|\Omega \right|}. \end{array}$$

From this, it follows that

$${∥\nabla u∥}_{2}\leq \frac{c\sqrt{\left|\Omega \right|}}{\sqrt{\lambda_{1}}\left(1-\left(\frac{a}{\lambda_{1}}+\frac{b}{\sqrt{\lambda_{1}\;}\;}\right)\right)}:=C_{0}.$$

Hence, the set of the solutions of Equation Family (17) is bounded in $H_{0}^{1}\left(\Omega \right)$. By the Leray–Schauder fixed-point theorem, A=S∘F has a fixed point $u_{0}\in H_{0}^{1}\left(\Omega \right)$, which belongs to $H^{2}\left(\Omega \right)$ and is an $L^{2}$ solution of BVP(1).

Next, we show that $u_{0}\in C^{2+\mu }\left(\bar{\Omega }\right)$, and it is a classical solution of BVP(1). Set

(19) $$h_{0}\left(x\right)=f\left(x,\;u_{0}\left(x\right),\;\nabla u_{0}\left(x\right)\right),\;x\in \Omega .$$

Then, $u_{0}=Sh_{0}$ is the solution of LBVP(13) for $h=h_{0}$.

If $p_{0}:=2<N$, choose $p_{1}=\frac{Np_{0}}{N-p_{0}}\left(>p_{0}\right)$; then, by the Sobolev embedding theorem, $H^{2}\left(\Omega \right)↪W^{1,p_{1}}\left(\Omega \right)$. Since $u_{0}\in H^{2}\left(\Omega \right)$, it follows that $u_{0}\in W^{1,p_{1}}\left(\Omega \right)$. By Condition (F3) and (19), we see that $h_{0}\in L^{p_{1}}\left(\Omega \right)$. Hence, by the existence and uniqueness of the $L^{p}$ solution of LBVP(13), $u_{0}=Sh_{0}\in W^{2,p_{1}}\left(\Omega \right)$.

If $p_{1}<N$, choose $p_{2}=\frac{Np_{1}}{N-p_{1}}\left(>p_{1}\right)$; then, by the Sobolev embedding theorem, $W^{2,p_{1}}\left(\Omega \right)↪W^{1,p_{2}}\left(\Omega \right)$. Hence, $u_{0}\in W^{1,p_{2}}\left(\Omega \right)$. By Condition (F3) and (19), we obtain that $h_{0}\in L^{p_{2}}\left(\Omega \right)$. Hence, $u_{0}=Sh_{0}\in W^{1,p_{2}}\left(\Omega \right)$.

To continue, since the step length $p_{k}-p_{k-1}\left(k=1,\;2,\;\cdots \right)$ is increasing, we can choose p>N such that $h_{0}\in L^{p}\left(\Omega \right)$. Thus, $u_{0}=Sh_{0}\in W^{2,p}\left(\Omega \right)$.

Choose $\sigma =\min\{1-\frac{N}{P},\;\mu \}$. By the Sobolev embedding theorem, $W^{2,p}\left(\Omega \right)↪C^{1+\sigma }\left(\bar{\Omega }\right)$. Hence, $u_{0}\in C^{1+\sigma }\left(\bar{\Omega }\right)$. By Assumption (F1)${}^{\prime }$ and (19), $h_{0}\in C^{\sigma }\left(\bar{\Omega }\right)$. Hence, by the Schauder theory of linear elliptic equations, the solution of LBVP(2.1) $u_{0}=Sh_{0}\in C^{2+\sigma }\left(\bar{\Omega }\right)$. By this and Assumption (F1)${}^{\prime }$, $h_{0}\in C^{\mu }\left(\bar{\Omega }\right)$. Hence, $u_{0}=Sh_{0}\in C^{2+\mu }\left(\bar{\Omega }\right)$. Clearly, $u_{0}$ is a classical solution of BVP(1). □

Strengthen Condition (F3) of Theorem 5; we have following existence and uniqueness result.

**Theorem** **6.** *Let $f:\bar{\Omega }\times R\times R^{N}\to R$ satisfy* (F1)${}^{\prime }$*, and for Following Condition*

(F4) *, there exist constants a,b≥0 satisfying (19) such that*
  $$\begin{array}{ccc} |f\left(x,\;\xi_{2},\;\eta_{2}\right)-f\left(x,\;\xi_{1},\;\eta_{1}\right)| & \leq & a|\xi_{2}-\xi_{1}\left|+b\right|\eta_{2}-\eta_{1}|,\\ & & \;x\in \bar{\Omega },\;\;\left(\xi_{1},\;\eta_{1}\right),\;\left(\xi_{2},\;\eta_{2}\right)\in \times R\times R^{N}. \end{array}$$

*Then, BVP(1) has at least one classical solution $u\in C^{2+\mu }\left(\bar{\Omega }\right)$.*

**Proof.**  We show that (F4)⟹(F3). Set $c=\max\{|f\left(x,\;0,\;0\right)|:\;x\in \bar{\Omega }\}+1$. For every $\left(x,\;\xi ,\;\eta \right)\in \bar{\Omega }\times R\times R^{N}$, by Condition (F4), we have

$$\begin{array}{ccc} \left|f(x,\;\xi ,\;\eta )\right| & \leq & \left|f(x,\;\xi ,\;\eta )-f(x,\;0,\;0)|+|f(x,\;0,\;0)\right|\\ & \leq & a|\xi |+b|\eta |+c. \end{array}$$

Hence, *f* satisfies Condition (F3). By Theorem 5, BVP(1) has at least one classical solution in $C^{2+\mu }\left(\bar{\Omega }\right)$.

Let $u_{1},\;u_{2}\in C^{2+\mu }\left(\bar{\Omega }\right)$ be the solutions of BVP(1). Set $u=u_{2}-u_{1}$ and $h=F\left(u_{2}\right)-F\left(u_{1}\right)$. By Assumption (F1)${}^{\prime }$, $h\in C^{\mu }\left(\bar{\Omega }\right)$. Since $u=u_{2}-u_{1}=Au_{2}-Au_{2}=S\left(F\left(u_{2}\right)\right)-S\left(F\left(u_{2}\right)\right)=Sh$, it follows that *u* is the clasical solution of LBVP(13). By (14) and Condition (F4), we have

$$\begin{array}{ccc} {∥\nabla u∥}_{2} & \leq & \frac{1}{\sqrt{\lambda_{1}}\;}{\;∥\Delta u∥}_{2}\;=\;\frac{1}{\sqrt{\lambda_{1}}\;}{\;∥h∥}_{2}\;=\;\frac{1}{\sqrt{\lambda_{1}}\;}{∥F\left(u_{2}\right)-F\left(u_{1}\right)∥}_{2}\\ & \leq & \frac{1}{\sqrt{\lambda_{1}}\;}\left(a∥u_{2}-u_{1}{∥}_{2}+b{∥\nabla u_{2}-\nabla u_{1}∥}_{2}\right)\\ & = & \frac{a}{\sqrt{\lambda_{1}}\;}{∥u∥}_{2}+\frac{b}{\sqrt{\lambda_{1}}\;}{∥\nabla u∥}_{2}\\ & \leq & \left(\frac{a}{\lambda_{1}}+\frac{b}{\sqrt{\lambda_{1}}\;}\right){∥\nabla u∥}_{2}. \end{array}$$

Since $\frac{a}{\lambda_{1}}+\frac{b}{\sqrt{\lambda_{1}}\;}<1$, from this inequality, it follows that ${∥\nabla u∥}_{2}=0$. By (14), ${∥u∥}_{2}=0$, and hence, $u_{1}=u_{2}$. This implies that BVP(1) has only one solution.

The proof of Theorem 6 is completed. □

**Theorem** **7.** *Let a,b≥0 and c>0 be constants, and $\frac{a}{\lambda_{1}}+\frac{b}{\sqrt{\lambda_{1}}\;}<1$. Then, the elliptic boundary value*

(20) $$\left\{\begin{array}{c} -\Delta u=a\;u+b\;|\nabla u|+c\;,\;x\in \Omega \;,\;\\ {u|}_{\partial \Omega }=0 \end{array}\right.$$

*has a unique positive classical solution $u\in C^{2+\mu }\left(\bar{\Omega }\right)\cap C^{+}\left(\bar{\Omega }\right)$.*

**Proof.**  Consider the elliptic boundary value

(21) $$\left\{\begin{array}{c} -\Delta u=a\;|u|+b\;|\nabla u|+c\;,\;x\in \Omega \;,\;\\ {u|}_{\partial \Omega }=0. \end{array}\right.$$

Corresponding to BVP(1), the nonlinearity *f* of BVP(21) is given by

(22) $$f\left(x,\;\xi ,\;\eta \right):=a\;|\xi |+b\;|\eta |+c,\;\left(x,\;\xi ,\;\eta \right)\in \bar{\Omega }\times R\times R^{N}.$$

It is easy to verify that the function *f* defined by (22) satisfies Conditions (F1)${}^{\prime }$ and (F4). Hence, by Theorem 6, BVP(21) has a unique solution $w_{0}\in C^{2+\mu }\left(\bar{\Omega }\right)$. Set

(23) $$h\left(x\right)=f\left(x,\;w_{0}\left(x\right),\;\nabla w_{0}\left(x\right)\right)),\;x\in \bar{\Omega };$$

then, $w_{0}$ is the classical solution of LBVP(13). Since $-\Delta w_{0}=h>0$, by the maximum principle of the elliptic operators, $w_{0}\left(x\right)>0$ for every x∈Ω. Hence, $w_{0}$ is a positive classical solution of BVP(20). On the other hand, the positive solution of BVP(20) is also a solution of BVP(21). By the uniqueness of the solution of BVP(21), $w_{0}$ is the unique positive classical solution of BVP(20). □

## 3. Proofs of the Main Results

**Proof** **of** **Theorem** **1.** Let a,b,H be the constants in the condition of Theorem 1. Choose a positive constant by

(24) $$c=\max\{|f\left(x,\;\xi ,\;\eta \right)|\;:\;\left(x,\;\xi ,\;\eta \right)\in \bar{\Omega }\times R\times R^{N},\;|\left(\xi ,\eta \right)|\leq H\;\}+1;$$

then, from Conditions (8) and (9), it follows that

(25) $$f\left(x,\;\xi ,\;\eta \right)\leq a\xi +b|\eta |+c,\;\left(x,\;\xi ,\;\eta \right)\in \bar{\Omega }\times R^{+}\times R^{N},$$

and

(26) $$f\left(x,-\xi ,-\eta \right)\geq -a\xi -b|\eta |-c,\;\left(x,\;\xi ,\;\eta \right)\in \bar{\Omega }\times R^{+}\times R^{N},$$

respectively. By Theorem 7, BVP(20) has a unique positive solution $w_{0}\in C^{2+\mu }\left(\bar{\Omega }\right)$. By Equation (20) and Inequality (25), we easily see that $w_{0}$ is an upper solution of BVP(1), and by (20) and (26), $-w_{0}$ is a lower solution of BVP(1). Since $-w_{0}\leq w_{0}$, by Theorem 4, BVP(1) has at least one solution $u\in C^{2+\mu }\left(\bar{\Omega }\right)$ between $-w_{0}$ and $w_{0}$. □

**Proof** **of** **Theorem** **2.** Let *c* be the positive constant defined by (24) and $w_{0}$ be the unique positive solution of BVP(20). Then by the proof of Theorem 1, $w_{0}\in C^{2+\mu }\left(\bar{\Omega }\right)$ is a upper solution of BVP(1).

It is well-known that the minimum positive real eigenvalue $\lambda_{1}$ of the elliptic eigenvalue problem

(27) $$\left\{\begin{array}{c} -\Delta u=\lambda \;u,\;x\in \Omega ,\\ {u|}_{\partial \Omega }=0, \end{array}\right.$$

has a positive unit eigenfunction; that is, there exists a function $\varphi_{1}\in C^{2}\left(\bar{\Omega }\right)\cap C^{+}\left(\bar{\Omega }\right)$ with $∥\varphi_{1}{∥}_{C(\bar{\Omega })}=1$ that satisfies the equation

(28) $$\left\{\begin{array}{c} -\Delta \varphi_{1}=\lambda_{1}\;\varphi_{1},\;x\in \Omega ,\\ \varphi_{1}{|}_{\partial \Omega }=0. \end{array}\right.$$

Let δ be the constant in (10), and choose

(29) $$\delta_{0}=\min\left\{\delta /{\left(1+{∥\nabla \varphi_{1}{∥}_{C}}^{2}\right)}^{1/2},\;c/\lambda_{1}\right\}.$$

Set $v_{0}=\delta_{0}\varphi_{1}$. By the regularity of the solution of the linear equation LBVP(13), $v_{0}\in C^{2+\mu }\left(\bar{\Omega }\right)$. For every $x\in \bar{\Omega }$, since $v_{0}\left(x\right)\geq 0$ and

(30) $$|\left(v_{0}\left(x\right),\;\nabla v_{0}\left(x\right)\right)|\leq \delta_{0}\;(|\varphi_{1}{\left(x\right)|}^{2}+|\nabla \varphi_{1}\left(x\right){{|}^{2})}^{1/2}\leq \delta_{0}\;{\left(1+{∥\nabla \varphi_{1}{∥}_{C(\bar{\Omega })}}^{2}\right)}^{1/2}\leq \delta ,$$

by the inequality (10) and Equation (28), we have

$$f\left(x,v_{0}\left(x\right),\nabla v_{0}\left(x\right)\right)\geq \lambda_{1}v_{0}\left(x\right)=-\Delta v_{0}\left(x\right).$$

Hence, $v_{0}$ is a lower solution of BVP(1). We show that $v_{0}\leq w_{0}$.

Consider the function $u=w_{0}-v_{0}$. Since $w_{0}$ satisfies Equation (20) and $v_{0}$ satisfies Equation (28), it follows that

$$-\Delta u\left(x\right)=a\;w_{0}\left(x\right)+b\;\left|\nabla w_{0}\left(x\right)\right|+c-\lambda_{1}v_{0}\left(x\right)\geq c-\lambda_{1}\delta_{0}\geq 0,\;x\in \Omega .$$

Since ${u|}_{\partial \Omega }=0$, by the maximum principle of the elliptic operators, u(x)>0 for every x∈Ω. Hence, $v_{0}\leq w_{0}$.

Therefore by Theorem 4, BVP(1) has at least one solution $u_{0}\in C^{2+\mu }\left(\bar{\Omega }\right)$ between $v_{0}$ and $w_{0}$ that is a positive solution of BVP(1). □

**Proof** **of** **Theorem** **3.** Let *c* be the positive constant defined by (24) and $w_{0}$ be the unique positive solution of BVP(20). Then by the proof of Theorem 1, $w_{0}$ is an upper solution and $-w_{0}$ is a lower solution of BVP(1).

Let $\delta_{0}$ be the positive constant defined by (29) and $v_{0}=\delta_{0}\varphi_{1}$. By the proof of Theorem 2, $v_{0}$ satisfies (30) and is a positive lower solution of BVP(1), and $v_{0}\leq w_{0}$. By the inequalities (30) and (12), we have

$$f\left(x,-v_{0}\left(x\right),-\nabla v_{0}\left(x\right)\right)\leq -\lambda_{1}v_{0}\left(x\right)=\Delta v_{0}\left(x\right)=-\Delta \left(-v_{0}\left(x\right)\right).$$

That is, $-v_{0}$ is an upper solution of BVP(1). Hence, we obtain two lower and upper solution pairs: $\left(v_{0},\;w_{0}\right)$ and $\left(-w_{0},\;-v_{0}\right)$, respectively. By Theorem 4, BVP(1) has one solution, $u_{1}$, between $v_{0}$ and $w_{0}$, and has another solution, $u_{2}$, between $-w_{0}$ and $-v_{0}$. Obviously, $u_{1}\geq v_{0}$ is positive and $u_{2}\leq -v_{0}$ is negative. □

**Example** **1.** *Consider the elliptic boundary value problem*

(31) $$\left\{\begin{array}{c} -\Delta u=a\;u-u^{2}+b\;|\nabla u|-{u|\nabla u|}^{2},\;x\in \Omega \;,\\ {u|}_{\partial \Omega }=0\;. \end{array}\right.$$

Clearly, 0 is a trivial solution of BVP(31). Let $a>\lambda_{1}$ and $b\leq \sqrt{\lambda_{1}}$. We use Theorem 2 to prove that BVP(31) has a positive classical solution. 

Corresponding to BVP(1), the nonlinearity of BVP(31) is

(32) $$f\left(x,\;\xi ,\;\eta \right)=a\xi -\xi^{2}+b|\eta |-{\xi |\eta |}^{2}.$$

Obviously, *f* satisfies (F1) and (F2). Choose

$$\varepsilon =\frac{1}{2}\;\frac{\lambda_{1}-\sqrt{\lambda_{1}}\;b}{1+\sqrt{\lambda_{1}}},\;a_{1}=\varepsilon ,\;b_{1}=b+\varepsilon ,\;H=\frac{\;a^{2}}{4\varepsilon }.$$

Then,

$$\frac{a_{1}}{\lambda_{1}}+\frac{b_{1}}{\sqrt{\lambda_{1}}}=\left(\frac{1}{\lambda_{1}}+\frac{1}{\sqrt{\lambda_{1}}}\right)\;\varepsilon +\frac{b}{\sqrt{\lambda_{1}}}<\left(\frac{1}{\lambda_{1}}+\frac{1}{\sqrt{\lambda_{1}}}\right)\;\frac{\lambda_{1}-\sqrt{\lambda_{1}}\;b}{1+\sqrt{\lambda_{1}}}+\frac{b}{\sqrt{\lambda_{1}}}=1;$$

when |(ξ,η)|>H, by (32), we have

$$\begin{array}{ccc} f(x,\;\xi ,\;\eta ) & \leq & a\xi -\xi^{2}+b|\eta |\;\leq \;\frac{a^{2}}{4}+b|\eta |=H\varepsilon +b|\eta |\\ & \leq & \varepsilon |(\xi ,\;\eta )|+b|\eta |\;\leq \;\varepsilon |\xi |+(\varepsilon +b)|\eta |\\ & = & a_{1}\left|\xi \right|+b_{1}\left|\eta \right|. \end{array}$$

Hence, *f* satisfies (8) for $a_{1}$ and $b_{1}$ restricted by (7). On the other hand, choose

$$\delta =\min\left\{1,\;\frac{a-\lambda_{1}}{2}\right\}.$$

Then, when ξ≥0 and |(ξ,η)|≤δ, by (32)

$$\begin{array}{ccc} f(x,\;\xi ,\;\eta ) & \geq & \lambda_{1}\xi +\xi (a-\lambda_{1}-\xi -{\left|\eta \right|}^{2})\\ & \geq & \lambda_{1}\xi +\xi (a-\lambda_{1}-\left|\left(\xi ,\;\eta \right)\right|-{\left|\left(\xi ,\;\eta \right)\right|}^{2})\\ & \geq & \lambda_{1}\xi +\xi (a-\lambda_{1}-2|\left(\xi ,\;\eta \right)|)\\ & \geq & \lambda_{1}\xi . \end{array}$$

Hence, *f* satisfies (10). By Theorem 2, BVP(31) has at least one positive solution. This result cannot be obtained from [14,15].

## Data Availability

Not applicable.
